# Parental and Caregiver Characteristics and Early Childhood Oral Health: A Cross-Sectional Study

**DOI:** 10.3390/healthcare14050580

**Published:** 2026-02-25

**Authors:** Rebeca Daniela Marton, Rahela Tabita Moca, Abel Emanuel Moca, Teofana Bota, Mihai Juncar

**Affiliations:** Department of Dentistry, Faculty of Medicine and Pharmacy, University of Oradea, 4 Universității Street, 410087 Oradea, Romania; rebeca.marton@uoradea.ro (R.D.M.); rahelamoca@gmail.com (R.T.M.); teofana.bota@uoradea.ro (T.B.); mihaijuncar@uoradea.ro (M.J.)

**Keywords:** early childhood oral health, parental sociodemographic factors, oral health behaviors, health literacy

## Abstract

Background/Objectives: Early childhood oral health is strongly influenced by parental and caregiver behaviors, yet evidence on the impact of sociodemographic factors remains limited in Eastern European settings. This study evaluated the association between parental and caregiver sociodemographic characteristics (age, educational level, and living environment) and oral health-related behaviors, dental attendance, and caries experience among preschool children in Romania. Methods: A cross-sectional study was conducted among parents or caregivers of children aged 0–6 years (*n* = 490). Data were collected between September and November 2025 using a structured online questionnaire (24 items). Statistical analyses included Fisher’s exact test, Mann–Whitney U test, Kruskal–Wallis test with post hoc analysis, and Spearman’s correlation (*p* < 0.05). Results: Higher parental education was consistently associated with favorable oral health behaviors, including earlier initiation of oral hygiene, parent-assisted toothbrushing, use of fluoridated toothpaste, and preventive dental visits (*p* < 0.01). Children of parents aged 21–30 years were more frequently caries-free (62.7%), whereas caries prevalence was higher among those with parents aged 41–50 years (60.5%) (*p* < 0.05). Urban residence was associated with twice-daily toothbrushing (49.4% vs. 36.2%) and earlier dental visits compared with rural residence (*p* < 0.05). Conclusions: Parental education, age, and living environment are significantly associated with early oral health behaviors and caries experience. Preventive programs should prioritize families with lower educational levels and those living in rural areas.

## 1. Introduction

In early childhood, oral health is largely associated with parental practices and behaviors rather than the child’s own choices [1]. At this stage, children lack autonomy with regard to diet, oral hygiene, and access to healthcare services, as these aspects depend almost entirely on the caregiving routines and behaviors adopted by adults within the family [2,3,4]. Consequently, early oral health often reflects the family environment in which the child grows and develops [5].

In practice, although the importance of prevention is widely recognized, recommendations are not always followed consistently [6]. The initiation of oral hygiene may be delayed, the consumption of sugary foods or beverages remains frequent, and the first dental visit often occurs at the onset of a problem rather than for preventive purposes [5,6]. These situations indicate that access to information does not automatically translate into stable preventive behaviors, as parental decisions are shaped by multiple factors more complex than knowledge alone [4]. Parental sociodemographic characteristics, particularly age and educational level, may be associated with how aspects related to a child’s oral health are managed [7]. Parental age may be related to caregiving style and level of experience, while educational attainment is often associated with the ability to understand and apply medical recommendations [4,5]. However, findings in the existing literature are not always consistent, and the relationship between these factors and children’s oral health remains insufficiently clarified.

These aspects are particularly relevant in relation to the onset and progression of dental caries in early childhood. Carious lesions may develop within the first years of life, often shortly after tooth eruption, and their progression can be rapid in the absence of appropriate preventive measures [8]. Beyond clinical consequences, early childhood caries (ECC) is associated with significant effects on both the child and the family, including pain, feeding difficulties, sleep disturbances, absenteeism from childcare or preschool, and the need for complex dental interventions [9,10]. Moreover, the emotional and financial burden experienced by families highlights the preventable nature of this condition and the importance of early identification of factors associated with its development [11,12].

However, the analysis of factors associated with early oral health needs to extend beyond the individual level, incorporating the social and organizational context in which parental decisions are made. Healthcare systems, access to dental services, and models of health education may be associated with differences in the relationship between parental sociodemographic characteristics and children’s oral care practices [13]. Most available studies originate from countries with well-structured healthcare systems and a strong tradition of preventive dentistry, whereas data from Eastern Europe remain limited [13]. In Romania, preventive dental services for young children are not systematically integrated into primary care, and important disparities exist in access to dental services and oral health education. Moreover, few studies from this context have simultaneously examined multiple parental sociodemographic characteristics in relation to a broad range of oral health-related behaviors, dental attendance patterns, and caries outcomes in preschool children. Addressing this gap may provide context-specific evidence to better understand social patterns and inequalities in early childhood oral health.

Therefore, the aim of the present study was to evaluate the association between parental and caregiver sociodemographic characteristics (such as age, educational level, and living environment) and oral health-related behaviors, access to dental services, and caries experience in preschool children. The study also examined whether these sociodemographic factors were associated with differences in preventive behaviors, dental attendance patterns, and caries outcomes in early childhood. It was hypothesized that lower educational level, older parental age, and rural residence would be associated with less favorable oral health-related behaviors and a higher prevalence of dental caries.

## 2. Materials and Methods

### 2.1. Study Design and Participants

This observational, cross-sectional study was conducted among parents or caregivers of children aged between 0 and 6 years. Data were collected between 1 September and 1 November 2025 using a structured questionnaire administered online via Google Forms (Google LLC, Mountain View, CA, USA). The questionnaire link was distributed through social media platforms and online groups dedicated to parents in order to facilitate access to the target population.

Each questionnaire was completed by a single parent or caregiver and referred to one child only. Responses were collected anonymously and analyzed in aggregate form.

Questionnaires completed by parents or caregivers of children under 6 years of age were included in the study. Questionnaires that were incomplete or did not refer to a child within the targeted age group were excluded from the analysis.

The study was reported in accordance with the Strengthening the Reporting of Observational Studies in Epidemiology (STROBE) guidelines.

### 2.2. Questionnaire and Variables

Data were collected on parental age (open-ended response), educational level (primary, lower secondary, upper secondary/vocational, post-secondary, university, and postgraduate education), and living environment (urban or rural).

Additional variables included child age, sex, number of siblings, children’s dietary habits, oral hygiene practices, dental attendance, caries experience, sources of information regarding the prevention of dental caries, and willingness to participate in educational sessions.

The research instrument was a structured questionnaire, developed in Romanian, consisting of 24 items and designed to assess factors associated with the oral health of children aged between 0 and 6 years. The questionnaire was organized into six distinct sections, each including items with predefined response options tailored to the type of information being assessed:

- Section I—Parental sociodemographic characteristics: parental age, educational level, and living environment;
- Section II—General information about the child: child age, sex, and number of siblings;
- Section III—Child’s diet: breastfeeding history, use of infant formula, consumption of sweetened beverages, falling asleep with a feeding bottle, and frequency of sweet food consumption;
- Section IV—Oral hygiene: timing of oral hygiene initiation, person responsible for toothbrushing, toothbrushing frequency, and use of fluoridated toothpaste;
- Section V—Dental history: previous dental attendance, age at the first dental visit, reason for the visit, presence of dental caries, and estimated number of carious lesions;
- Section VI—Additional information: sources of information regarding the prevention of dental caries and willingness to participate in educational sessions.

The questionnaire was developed based on previously published studies and current recommendations regarding early childhood oral health. To ensure content validity, the instrument was reviewed by two senior specialists in pediatric dentistry. Prior to data collection, the questionnaire was pretested on a small group of 15 parents to assess clarity and comprehensibility, and minor adjustments were made based on their feedback.

Each questionnaire item was analyzed individually rather than combined into a composite score. This approach was chosen to preserve the specificity of individual behaviors and practices, as the included items reflected distinct dimensions of children’s oral health (dietary habits, oral hygiene, and dental attendance). Given the exploratory nature of the study and the absence of a previously validated composite index for the investigated variables, no overall behavioral score was constructed.

As the questionnaire was designed to collect information across multiple independent domains rather than to measure a single latent construct, internal consistency analysis was not considered appropriate. Applying such measures to heterogeneous variables could have resulted in misleading interpretations. Therefore, the data were analyzed and interpreted at the level of individual items.

### 2.3. Sample Size

The minimum required sample size was estimated a priori for a cross-sectional study using Python 3.10 (Python Software Foundation, Wilmington, DE, USA). The calculation assumed a 95% confidence level and a margin of error of 5%. In the absence of prior estimates for the proportion of the variables of interest, a conservative proportion of *p* = 0.5 was applied in order to ensure an adequately sized sample, as this value yields the maximum variability and therefore the largest required sample size. Based on these assumptions, the minimum estimated sample size was approximately 384 participants. After applying the inclusion and exclusion criteria, the final sample consisted of 490 valid questionnaires.

### 2.4. Statistical Analysis

Statistical analysis was performed using IBM SPSS Statistics, version 25 (IBM Corp., Armonk, NY, USA). Microsoft Excel 2025 and Microsoft Word 2025 (Microsoft Corp., Redmond, WA, USA) were used for data organization, initial data processing, and presentation of the results.

Quantitative variables were tested for normality using the Shapiro–Wilk test, with *p* < 0.05 indicating a non-normal distribution. Depending on the distribution, continuous variables are expressed as means with standard deviations or medians with interquartile ranges. Categorical variables are expressed as absolute values and percentages.

Comparisons between categorical variables were performed using Fisher’s exact test. To further explore significant associations identified in contingency tables, Z-tests with Bonferroni correction were applied in order to control for type I error in multiple comparisons.

Non-normally distributed quantitative variables were compared between two groups using the Mann–Whitney U test and among more than two groups using the Kruskal–Wallis H test. When statistically significant differences were observed, Dunn’s post hoc tests with Bonferroni correction were conducted. Correlations between non-normally distributed quantitative variables were assessed using Spearman’s rank correlation coefficient (rho).

Given the exploratory nature of the study and the categorical structure of several variables, with some small subgroup sizes, the analysis focused on bivariate methods to describe associations between parental sociodemographic characteristics and oral health outcomes. Multivariable models were not applied, as the distribution of observations across multiple categories could have resulted in unstable estimates.

The level of statistical significance was set at α = 0.05.

### 2.5. Ethical Considerations

The study was conducted in accordance with the ethical principles outlined in the Declaration of Helsinki (1964) and its subsequent amendments. The research protocol was approved by the Ethics Committee of the University of Oradea (IRB No. 9970/23.06.2025). Participation in the study was voluntary and anonymous. Prior to completing the questionnaire, respondents were informed about the purpose of the research and the exclusive scientific use of the collected data. No financial or other incentives were provided. Informed consent was implied through the voluntary completion of the questionnaire.

## 3. Results

Table 1 summarizes the background characteristics of the study population. A total of 490 parents or caregivers were included in the analysis. Most respondents were aged 31–40 years (56.5%). The majority had higher education, with university (41.0%) and postgraduate education (24.9%) being the most common levels. Most participants lived in urban areas (69%).

The mean age of the children was 4.09 ± 1.73 years (median: 4.13 years). The sex distribution was nearly balanced, with a slight predominance of males (50.2%). Regarding family structure, 27.8% of the children had no siblings, while the largest proportion had one sibling (34.3%). The mean number of siblings was 1.33 ± 1.19 (median: 1).

Table 2 presents the distribution of respondents according to parental age and questionnaire items. Several significant associations were observed between parental age and children’s feeding practices, oral hygiene behaviors, dental attendance, and caries-related outcomes.

Breastfeeding prevalence and duration differed across parental age groups, being more common and longer among parents aged 31–40 and 41–50 years. In addition, the absence of infant formula use and avoidance of bottle use at bedtime were more frequently reported in this group (*p* < 0.05).

Dietary habits also varied according to parental age. Lower consumption of sweet foods was reported among children of the youngest parents, whereas more frequent intake was observed among children of parents aged 41–50 years (*p* < 0.001).

Differences were also identified in oral hygiene practices. Lack of toothbrushing was more frequently reported among children of parents younger than 20 years, while parent-assisted toothbrushing was more common among those aged 21–30 years (*p* = 0.010).

Dental attendance and caries-related outcomes showed significant variation across parental age groups. Children of older parents were more likely to have had a dental visit and to be reported with dental caries, whereas caries-free status and earlier detection of lesions were more frequently reported among children of younger parents (*p* < 0.05). These findings should be interpreted with caution, as differences in children’s age distribution and the self-reported nature of the data may have influenced the observed patterns.

Significant differences in oral hygiene practices and dental attendance were observed according to parents’ area of residence. Children of parents living in rural areas more frequently brushed their teeth once per day, whereas brushing twice or more per day was more common among children of parents residing in urban areas (*p* = 0.041). Dental attendance was significantly higher among children of urban parents compared with those from rural areas (*p* = 0.020). Additionally, children of urban parents had their first dental visit at a significantly younger age than those of rural parents (*p* < 0.001) (Table 3).

Table 4 presents the distribution of respondents according to parental education level and questionnaire items. Significant differences were observed across education groups in relation to children’s feeding practices, oral hygiene behaviors, dental attendance, and caries-related outcomes.

Breastfeeding prevalence and duration increased significantly with higher parental education levels, being more frequent and longer among parents with university and postgraduate education compared with those with lower education levels (*p* < 0.001). The consumption of sweetened liquids, bedtime bottle use, and the frequency of sweet food intake also differed significantly by parental education, indicating less favorable dietary behaviors among children of parents with lower educational attainment (*p* < 0.05).

Oral hygiene practices were strongly associated with parental education. Earlier initiation of oral hygiene and joint parent–child toothbrushing were more common among parents with university and postgraduate education, whereas delayed or absent oral hygiene practices were more frequent among parents with primary or lower secondary education (*p* < 0.001). The use of fluoride toothpaste was also significantly associated with higher parental education levels, while uncertainty regarding fluoride use was more common among parents with lower education (*p* < 0.001).

Dental attendance patterns varied significantly by parental education. Children of parents with university and postgraduate education were more likely to have visited a dentist and to have had their first dental visit at a younger age compared with those whose parents had lower educational levels (*p* < 0.01). Preventive visits were more frequent among children of highly educated parents, whereas visits prompted by dental pain were more common among children of parents with lower education (*p* = 0.001).

Finally, parental education was significantly associated with awareness of caries prevention and willingness to participate in educational sessions. Parents with higher education levels more frequently reported receiving information from dental professionals or multiple sources and expressed greater interest in participating in oral health information sessions (*p* < 0.01).

## 4. Discussion

The findings of the present study highlight the central role of parental sociodemographic characteristics in shaping oral health-related behaviors among preschool children. In our study, parental age, educational level, and living environment were significantly associated with dietary practices, oral hygiene behaviors, access to dental services, and children’s caries experience, highlighting the extent to which early oral health is strongly influenced by the family and social context.

One of the most consistent findings of the present study was the association between parental educational level and preventive behaviors. Parents with university and postgraduate education more frequently reported early initiation of oral hygiene, toothbrushing performed together with the child, use of fluoridated toothpaste, and dental visits at younger ages, predominantly for preventive purposes. These findings are in line with the international literature, which identifies educational level as a major determinant of health literacy and of the ability to understand and apply medical recommendations [14,15]. Higher educational levels are associated not only with greater knowledge but also with more favorable attitudes towards prevention and a clearer perception of the long-term risks associated with neglecting oral health during early childhood [4,14,15]. Similar to our results, Popovic et al. reported that children’s oral hygiene behaviors, including the timing of the first dental visit and parental involvement in toothbrushing, were significantly associated with maternal education, which emerged as one of the most consistent predictors of children’s oral hygiene practices [16]. In the same direction, a recent study conducted among preschool children showed that higher maternal educational level increased the likelihood of supervised toothbrushing, use of fluoridated toothpaste, and dental visits within the previous year, suggesting that parental education translates into concrete preventive practices at the family level [17].

In contrast, parents with lower educational levels more frequently reported cariogenic dietary behaviors, delayed initiation or absence of oral hygiene practices, and dental visits primarily motivated by pain or acute problems. This pattern suggests the presence of multiple barriers, which may include limited access to high-quality health information, prioritization of other needs perceived as more urgent, as well as previous negative experiences with dental services [18]. Consistent with the findings of the present study, recent evidence indicates that lower maternal educational level is associated with a higher risk of cariogenic dietary intake, poorer oral hygiene practices, and reduced utilization of preventive dental services, highlighting the direct influence of parental education on children’s dietary and oral care behaviors. These findings suggest that lower educational levels may contribute to less healthy dietary habits and reduced engagement in preventive practices at the family level [19].

Parental age emerged as another relevant factor, influencing both preventive behaviors and children’s caries experience. Younger parents (21–30 years) more frequently reported caries-free children and earlier detection of carious lesions, whereas children of older parents (41–50 years) exhibited a higher prevalence of dental caries. These differences may reflect generational changes in access to information, exposure to public health messages, and attitudes towards prevention [20]. Younger parents may be more receptive to contemporary preventive recommendations and more familiar with digital sources of health information, while older parents may tend to reproduce more traditional oral care models that are less oriented towards early prevention [20,21]. In the literature, the association between parental age (particularly maternal age) and children’s caries risk has been reported inconsistently. While some studies identify younger maternal age as a risk factor, potentially reflecting more limited resources or parental support, others report a higher caries risk among children of older mothers or describe a U-shaped relationship, with increased risk at both younger and older maternal ages compared with intermediate age groups [22,23,24].

The differences observed between urban and rural settings confirm the existence of inequalities in access to dental services and in oral health-related behaviors. Children living in urban areas more frequently benefited from twice-daily toothbrushing and from dental visits at younger ages compared with their rural counterparts. These findings are particularly relevant in the Romanian context, where preventive dental services for children are not systematically integrated into primary healthcare and where the distribution of dental practices is uneven, with a clear predominance in urban areas [25,26,27]. In addition, Romania does not have national fluoride fortification programs, such as community water fluoridation or salt or milk fluoridation. As a result, fluoride exposure depends mainly on individual preventive measures, including the use of fluoridated toothpaste and professional topical applications [28]. Limited infrastructure, associated costs, and greater distances to specialized services may further contribute to delayed access to dental care in rural settings [29]. Similar patterns have been reported in other Central and Eastern European countries, including Poland, Bulgaria, and Hungary, where studies indicate persistent urban–rural disparities in access to preventive dental services, toothbrushing frequency, and age at first dental visit. These findings suggest that such inequalities are closely linked to healthcare system organization, the distribution of service providers, and the extent to which oral health prevention is integrated into primary care [30,31,32].

An important finding of the present study is that, although a substantial proportion of parents reported having received information on caries prevention, this did not consistently translate into adequate preventive behaviors. This discrepancy suggests that oral health education interventions need to move beyond an information-based model and incorporate interactive, family-centered strategies that take into account the real-life constraints faced by parents [33]. Active involvement of the dentist in early oral health education, starting from the eruption of the first teeth, may have a significant impact on reducing the incidence of early childhood caries [34].

The results of this study should be interpreted considering several limitations. The cross-sectional design does not allow causal relationships to be established, and the data were based on parental self-report, which may be subject to recall bias or social desirability bias. In addition, the statistical analysis relied on bivariate methods only, and no multivariable adjustment was performed. Parental sociodemographic characteristics such as age, educational level, and living environment are likely interrelated; therefore, their independent effects on children’s oral health outcomes could not be assessed, and the observed associations should be interpreted as unadjusted relationships that may be influenced by confounding.

The representativeness of the study sample should also be considered. Compared with the general Romanian population, the sample included a higher proportion of urban residents and parents with higher educational levels, most likely due to the online recruitment strategy and voluntary participation. This selection pattern may have favored the inclusion of parents with greater access to digital resources and a higher interest in health-related topics, potentially leading to an overestimation of preventive behaviors and health awareness. Consequently, the findings should be interpreted with caution and cannot be considered fully representative of the national population. Nevertheless, the relatively large sample size and the detailed analysis of multiple sociodemographic characteristics provide a relevant overview of patterns and inequalities related to early childhood oral health within the Romanian context.

## 5. Conclusions

This study showed that parental educational level was the factor most consistently associated with children’s oral health-related behaviors, including earlier initiation of oral hygiene, use of fluoridated toothpaste, parent-assisted toothbrushing, and preventive dental attendance. Parental age was also associated with feeding practices, oral hygiene behaviors, and caries experience, while urban residence was related to more frequent toothbrushing and earlier dental visits.

These findings indicate that early childhood oral health in the studied population is strongly related to parental sociodemographic characteristics. Targeted preventive strategies should therefore prioritize families with lower educational levels and those living in rural areas in order to reduce inequalities in early oral health.

## Figures and Tables

**Table 1:** Distribution of respondents according to the characteristics analyzed in the study (*n* = 490).

Section	Answer	Value
**Section I—Parental Sociodemographic Characteristics**	**Item 1—“What is your age?”**	
	Under 20 years	4 (0.8%)
	21–30 years	166 (33.9%)
	31–40 years	277 (56.5%)
	41–50 years	43 (8.8%)
	**Item 2—“What is the highest level of education you have completed?”**	
	Primary	2 (0.4%)
	Lower secondary	8 (1.6%)
	Upper secondary	72 (14.7%)
	Post-secondary	85 (17.3%)
	University	201 (14%)
	Postgraduate	122 (24.9%)
	**Item 3—“What is your living environment?”**	
	Rural	152 (31%)
	Urban	338 (69%)
**Section II—General Information about the Child**	**Item 4—“What is your child’s age?”**	
	Mean ± SD	4.09 ± 1.73
	Median (IQR)	4.13 (2.58–5.85)
	**Item 5—“What is your child’s sex?”**	
	Female	244 (49.8%)
	Male	246 (50.2%)
	**Item 6—“How many siblings does your child have?”**	
	None	136 (27.8%)
	1 sibling	168 (34.3%)
	2 siblings	111 (22.7%)
	3 siblings	47 (9.6%)
	4 siblings	19 (3.9%)
	5 siblings	9 (1.8%)

**Table 2:** Distribution of respondents according to parental age and questionnaire items (*n* = 490).

Section	Answers	Age				*p*
		<20 Years	21–30 Years	31–40 Years	41–50 Years	
**Section III—Child’s Diet**	**Item 7—“Was the child breastfed?”**					
	No	2 (50.0%)	33 (19.9%)	32 (11.6%)	6 (14.0%)	0.021
	Yes	2 (50.0%)	133 (80.1%)	245 (88.4%)	37 (86.0%)	
	**Item 8—“If yes, until what age?”**					
	Mean ± SD	0.71 ± 0.41	1.12 ± 0.8	1.47 ± 0.93	1.66 ± 1.14	0.001
	Median (IQR)	0.7 (0.4–1)	1 (0.5–1.5)	1.42 (0.58–2)	1.5 (0.67–2.5)	
	**Item 9—“Does or did the child receive infant formula?”**					
	No	1 (25.0%)	65 (39.2%)	148 (53.4%)	24 (55.8%)	0.042
	Occasionally	0 (0.0%)	24 (14.5%)	27 (9.7%)	2 (4.7%)	
	Yes	3 (75.0%)	77 (46.4%)	102 (36.8%)	17 (39.5%)	
	**Item 10—“Does the child consume sweetened liquids?”**					
	No	3 (75.0%)	45 (27.1%)	62 (22.4%)	7 (16.3%)	0.233
	Occasionally	1 (25.0%)	76 (45.8%)	133 (48.0%)	20 (46.5%)	
	Yes	0 (0.0%)	45 (27.1%)	82 (29.6%)	16 (37.2%)	
	**Item 11—“Does the child frequently fall asleep with a bottle in their mouth?”**					
	No	3 (75.0%)	136 (81.9%)	252 (91%)	38 (88.4%)	0.026
	Occasionally	0 (0.0%)	16 (9.6%)	8 (2.9%)	1 (2.3%)	
	Yes	1 (25.0%)	14 (8.4%)	17 (6.1%)	4 (9.3%)	
	**Item 12—“How often does the child consume sweet foods?”**					
	Never	3 (75.0%)	31 (18.7%)	28 (10.1%)	1 (2.3%)	<0.001
	Rarely	0 (0.0%)	44 (26.5%)	72 (26%)	15 (34.9%)	
	Often	1 (25.0%)	67 (40.4%)	110 (39.7%)	13 (30.2%)	
	Very often	0 (0.0%)	24 (14.5%)	67 (24.2%)	14 (32.6%)	
**Section IV—Oral Hygiene**	**Item 13—“At what age did the child start oral hygiene practices?”**					
	Before 1 year	0 (0.0%)	60 (36.1%)	132 (47.7%)	18 (41.9%)	0.083
	Between 1–2 years	3 (75.0%)	78 (47.0%)	102 (36.8%)	19 (44.2%)	
	After 2 years	0 (0.0%)	22 (13.3%)	36 (13.0%)	6 (14.0%)	
	Never	1 (25.0%)	6 (3.6%)	7 (2.5%)	0 (0.0%)	
	**Item 14—“Who brushes the child’s teeth?”**					
	No one	1 (25.0%)	6 (3.6%)	7 (2.5%)	0 (0.0%)	0.010
	Only the parent	2 (50.0%)	33 (19.9%)	30 (10.8%)	4 (9.3%)	
	Both parent and child together	1 (25.0%)	99 (59.6%)	184 (66.4%)	26 (60.5%)	
	Only the child	0 (0.0%)	28 (16.9%)	56 (20.2%)	13 (30.2%)	
	**Item 15—“How many times per day does the child brush their teeth?”**					
	Never	1 (25.0%)	6 (3.6%)	7 (2.5%)	0 (0.0%)	0.299
	Less than once a day	0 (0.0%)	10 (6.0%)	20 (7.2%)	3 (7.0%)	
	Once a day	2 (50.0%)	77 (46.4%)	128 (46.2%)	14 (32.6%)	
	Two times or more a day	1 (25.0%)	73 (44.0%)	122 (44.0%)	26 (60.5%)	
	**Item 16—“Is fluoride toothpaste used for the child?”**					
	No	1 (25.0%)	34 (20.5%)	62 (22.4%)	9 (20.9%)	0.392
	Yes	3 (75.0%)	108 (65.1%)	192 (69.3%)	32 (74.4%)	
	I don’t know	0 (0.0%)	24 (14.5%)	23 (8.3%)	2 (4.7%)	
**Section V—Dental History**	**Item 17—“Has the child ever visited a dentist?”**					
	No	3 (75.0%)	76 (45.8%)	65 (23.5%)	4 (9.3%)	<0.001
	Yes	1 (25.0%)	90 (54.2%)	212 (76.5%)	39 (90.7%)	
	**Item 18—“If yes, at what age was the first visit?”**					
	Mean ± SD	2	2.77 ± 1.33	3.06 ± 1.57	3.46 ± 2.26	0.330
	Median (IQR)	2	2.58 (2–4)	3 (2–4)	3 (2–4.5)	
	**Item 19—“What was the main reason for the first visit?”**					
	Check-up	1 (100.0%)	53 (58.9%)	147 (69.3%)	27 (69.2%)	0.522
	Frontal teeth caries	0 (0.0%)	9 (10%)	16 (7.5%)	1 (2.6%)	
	Dental pain	0 (0.0%)	19 (21.1%)	36 (17.0%)	8 (20.5%)	
	Dental trauma	0 (0.0%)	5 (5.6%)	11 (5.2%)	3 (7.7%)	
	Posterior teeth caries	0 (0.0%)	3 (3.3%)	1 (0.5%)	0 (0.0%)	
	Extraction	0 (0.0%)	1 (1.1%)	0 (0.0%)	0 (0.0%)	
	Other surgical intervention	0 (0.0%)	0 (0.0%)	1 (0.5%)	0 (0.0%)	
	**Item 20—“Has your child had dental caries so far?”**					
	No	4 (100.0%)	104 (62.7%)	153 (55.2%)	16 (37.2%)	0.029
	Yes	0 (0.0%)	58 (34.9%)	119 (43%)	26 (60.5%)	
	I don’t know	0 (0.0%)	4 (2.4%)	5 (1.8%)	1 (2.3%)	
	**Item 21—“If yes, at what age were the first caries identified?”**					
	Mean ± SD	-	3.48 ± 1.14	3.7 ± 1.42	4.25 ± 1	0.027
	Median (IQR)	-	3 (2.98–4)	3.5 (3–5)	4 (3.75–5)	
	**Item 22—“How many carious lesions does your child currently have?”**					
	I don’t know	0 (0.0%)	8 (4.8%)	14 (5.1%)	2 (4.7%)	0.550
	None	4 (100.0%)	115 (69.3%)	183 (66.1%)	28 (65.1%)	
	1–3	0 (0.0%)	30 (18.1%)	64 (23.1%)	12 (27.9%)	
	4–6	0 (0.0%)	11 (6.6%)	15 (5.4%)	0 (0.0%)	
	More than 6	0 (0.0%)	2 (1.2%)	1 (0.4%)	1 (2.3%)	
	Yes	4 (100%)	134 (80.7%)	220 (79.4%)	30 (69.8%)	
**Section VI—Additional Information**	**Item 23—“Have you received information about the prevention of dental caries in children?”**					
	No	0 (0.0%)	23 (13.9%)	26 (9.4%)	0 (0.0%)	0.021
	Yes, from other sources	2 (50.0%)	41 (24.7%)	52 (18.8%)	6 (14.0%)	
	Yes, from the dentist	2 (50.0%)	87 (52.4%)	169 (61.0%)	35 (81.4%)	
	Yes, from both	0 (0.0%)	15 (9.0%)	30 (10.8%)	2 (4.7%)	
	**Item 24—“Would you like to participate in a short information session on children’s oral health?”**					
	No	0 (0.0%)	32 (19.3%)	57 (20.6%)	13 (30.2%)	0.370
	Yes	4 (100.0%)	134 (80.7%)	220 (79.4%)	30 (69.8%)	

**Table 3:** Distribution of respondents according to parental living environment and questionnaire items (*n* = 490).

Section	Answers	Living Environment		*p*
		Rural	Urban	
**Section III—Child’s Diet**	**Item 7—“Was the child breastfed?”**			
	No	26 (17.1%)	47 (13.9%)	0.411
	Yes	126 (82.9%)	291 (86.1%)	
	**Item 8—“If yes, until what age?”**			
	Mean ± SD	1.25 ± 0.88	1.42 ± 0.94	0.100
	Median (IQR)	1 (0.5–2)	1.25 (0.5–2)	
	**Item 9—“Does or did the child receive infant formula?”**			
	No	82 (53.9%)	156 (46.2%)	0.254
	Occasionally	13 (8.6%)	40 (11.8%)	
	Yes	57 (37.5%)	142 (42%)	
	**Item 10—“Does the child consume sweetened liquids?”**			
	No	34 (22.4%)	83 (24.6%)	0.811
	Occasionally	71 (46.7%)	159 (47.0%)	
	Yes	47 (30.9%)	96 (28.4%)	
	**Item 11—“Does the child frequently fall asleep with a bottle in their mouth?”**			
	No	127 (83.6%)	302 (89.3%)	0.148
	Occasionally	9 (5.9%)	16 (4.7%)	
	Yes	16 (10.5%)	20 (5.9%)	
	**Item 12—“How often does the child consume sweet foods?”**			
	Never	22 (14.5%)	41 (12.1%)	0.880
	Rarely	39 (25.7%)	92 (27.2%)	
	Often	60 (39.5%)	131 (38.8%)	
	Very often	31 (20.4%)	74 (21.9%)	
**Section IV—Oral Hygiene**	**Item 13—“At what age did the child start oral hygiene practices?”**			
	Before 1 year	56 (36.8%)	154 (45.6%)	0.168
	Between 1–2 years	65 (42.8%)	137 (40.5%)	
	After 2 years	26 (17.1%)	38 (11.2%)	
	Never	5 (3.3%)	9 (2.7%)	
	**Item 14—“Who brushes the child’s teeth?”**			
	No one	5 (3.3%)	9 (2.7%)	0.061
	Only the parent	23 (15.1%)	46 (13.6%)	
	Both parent and child together	84 (55.3%)	226 (66.9%)	
	Only the child	40 (26.3%)	57 (16.9%)	
	**Item 15—“How many times per day does the child brush their teeth?”**			
	Never	5 (3.3%)	9 (2.7%)	0.041
	Less than once a day	10 (6.6%)	23 (6.8%)	
	Once a day	82 (53.9%)	139 (41.1%)	
	Two times or more a day	55 (36.2%)	167 (49.4%)	
	**Item 16—“Is fluoride toothpaste used for the child?”**			
	No	30 (19.7%)	76 (22.5%)	0.277
	Yes	102 (67.1%)	233 (68.9%)	
	I don’t know	20 (13.2%)	29 (8.6%)	
**Section V—Dental History**	**Item 17—“Has the child ever visited a dentist?”**			
	No	57 (37.5%)	91 (26.9%)	0.020
	Yes	95 (62.5%)	247 (73.1%)	
	**Item 18—“If yes, at what age was the first visit?”**			
	Mean ± SD	3.55 ± 1.78	2.83 ± 1.5	<0.001
	Median (IQR)	3 (2–4.33)	3 (2–4)	
	**Item 19—“What was the main reason for the first visit?”**			
	Check-up	61 (64.2%)	167 (67.6%)	0.747
	Frontal teeth caries	9 (9.5%)	17 (6.9%)	
	Dental pain	18 (18.9%)	45 (18.2%)	
	Dental trauma	5 (5.3%)	14 (5.7%)	
	Posterior teeth caries	1 (1.1%)	3 (1.2%)	
	Extraction	1 (1.1%)	0 (0.0%)	
	Other surgical intervention	0 (0.0%)	1 (0.4%)	
	**Item 20—“Has your child had dental caries so far?”**			
	No	87 (57.2%)	190 (56.2%)	0.423
	Yes	64 (42.1%)	139 (41.1%)	
	I don’t know	1 (0.7%)	9 (2.7%)	
	**Item 21—“If yes, at what age were the first caries identified?”**			
	Mean ± SD	3.94 ± 1.17	3.6 ± 1.36	0.132
	Median (IQR)	4 (3–5)	3.5 (3–5)	
	**Item 22—“How many carious lesions does your child currently have?”**			
	I don’t know	7 (4.6%)	17 (5.0%)	0.827
	None	98 (64.5%)	232 (68.6%)	
	1–3	36 (32.7%)	70 (20.7%)	
	4–6	10 (6.6%)	16 (4.7%)	
	More than 6	1 (0.7%)	3 (0.9%)	
**Section VI—Additional Information**	**Item 23—“Have you received information about the prevention of dental caries in children?”**			
	No	16 (10.5%)	33 (9.8%)	0.252
	Yes, from other sources	39 (25.7%)	62 (18.3%)	
	Yes, from the dentist	82 (53.9%)	211 (62.4%)	
	Yes, from both	15 (9.9%)	32 (9.5%)	
	**Item 24—“Would you like to participate in a short information session on children’s oral health?”**			
	No	27 (17.8%)	75 (22.2%)	0.281
	Yes	125 (82.2%)	263 (77.8%)	

**Table 4:** Distribution of respondents according to parental education level and questionnaire items (*n* = 490).

Section	Answers	Education Level						*p*
		Primary	Lower Secondary	Upper Secondary	Post-Secondary	University	Postgraduate	
**Section III—Child’s Diet**	**Item 7—“Was the child breastfed?”**							
	No	2 (100.0%)	1 (12.5%)	16 (22.2%)	20 (23.5%)	29 (14.4%)	5 (4.1%)	<0.001
	Yes	0 (0.0%)	7 (87.5%)	56 (77.8%)	65 (76.5%)	172(85.6%)	117 (95.9%)	
	**Item 8—“If yes, until what age?”**							
	Mean ± SD	-	2.02 ± 1.15	1.07 ± 0.84	0.94 ± 0.66	1.46 ± 0.9	1.58 ± 1	<0.001
	Median (IQR)	-	2.5 (1–3)	0.9 (0.3–1.6)	0.8 (0.4–1.2)	1.5 (0.6–2)	1.42 (0.8–2.08)	
	**Item 9—“Does or did the child receive infant formula?”**							
	No	0 (0.0%)	4 (50.0%)	28 (38.9%)	35 (41.2%)	104(51.7%)	67 (54.9%)	0.182
	Occasionally	0 (0.0%)	1 (12.5%)	9 (12.5%)	10 (11.8%)	17 (8.5%)	16 (13.1%)	
	Yes	2 (100.0%)	3 (37.5%)	35 (48.6%)	40 (47.1%)	80 (39.8%)	39 (32.0%)	
	**Item 10—“Does the child consume sweetened liquids?”**							
	No	0 (0.0%)	0 (0.0%)	15 (20.8%)	30 (35.3%)	48 (23.9%)	24 (19.7%)	<0.001
	Occasionally	1 (50.0%)	4 (50.0%)	30 (41.7%)	47 (55.3%)	93 (46.3%)	55 (45.1%)	
	Yes	1 (50.0%)	4 (50.0%)	27 (37.5%)	8 (9.4%)	60 (29.9%)	43 (35.2%)	
	**Item 11—“Does the child frequently fall asleep with a bottle in their mouth?”**							
	No	2 (100.0%)	8 (100.0%)	58 (80.6%)	66 (77.6%)	181 (90%)	114 (93.4%)	0.044
	Occasionally	0 (0.0%)	0 (0.0%)	5 (6.9%)	9 (10.6%)	9 (4.5%)	2 (1.6%)	
	Yes	0 (0.0%)	0 (0.0%)	9 (12.5%)	10 (11.8%)	11 (5.5%)	6 (4.9%)	
	**Item 12—“How often does the child consume sweet foods?”**							
	Never	0 (0.0%)	0 (0.0%)	11 (15.3%)	21 (24.7%)	24 (11.9%)	7 (5.7%)	<0.001
	Rarely	1 (50.0%)	0 (0.0%)	11 (15.3%)	41 (48.2%)	46 (22.9%)	32 (26.2%)	
	Often	1 (50.0%)	3 (37.5%)	36 (50.0%)	17 (20.0%)	88 (43.8%)	46 (37.7%)	
	Very often	0 (0.0%)	5 (62.5%)	14 (19.4%)	6 (7.1%)	43 (21.4%)	37 (30.3%)	
**Section IV—Oral Hygiene**	**Item 13—“At what age did the child start oral hygiene practices?”**							
	Before 1 year	0 (0.0%)	2 (25.0%)	15 (20.8%)	17 (20.0%)	101(50.2%)	75 (61.5%)	<0.001
	Between 1–2 years	0 (0.0%)	1 (12.5%)	43 (59.7%)	45 (52.9%)	81 (40.3%)	32 (26.2%)	
	After 2 years	2 (100.0%)	5 (62.5%)	12 (16.7%)	19 (22.4%)	14 (7.0%)	12 (9.8%)	
	Never	0 (0.0%)	0 (0.0%)	2 (2.8%)	4 (4.7%)	5 (2.5%)	3 (2.5%)	
	**Item 14—“Who brushes the child’s teeth?”**							
	No one	0 (0.0%)	0 (0.0%)	2 (2.8%)	4 (4.7%)	5 (2.5%)	3 (2.5%)	0.005
	Only the parent	2 (100.0%)	0 (0.0%)	13 (18.1%)	11 (12.9%)	30 (14.9%)	13 (10.7%)	
	Both parent and child together	0 (0.0%)	4 (50.0%)	44 (61.1%)	42 (49.4%)	129(64.2%)	91 (74.6%)	
	Only the child	0 (0.0%)	4 (50.0%)	13 (18.1%)	28 (32.9%)	37 (18.4%)	15 (12.3%)	
	**Item 15—“How many times per day does the child brush their teeth?”**							
	Never	0 (0.0%)	0 (0.0%)	2 (2.8%)	4 (4.7%)	5 (2.5%)	3 (2.5%)	0.097
	Less than once a day	0 (0.0%)	1 (12.5%)	3 (4.2%)	1 (1.2%)	15 (7.5%)	13 (10.7%)	
	Once a day	2 (100.0%)	6 (75.0%)	42 (58.3%)	32 (37.6%)	84 (41.8%)	55 (45.1%)	
	Two times or more a day	0 (0.0%)	1 (12.5%)	25 (34.7%)	48 (56.5%)	97 (48.3%)	51 (41.8%)	
	**Item 16—“Is fluoride toothpaste used for the child?”**							
	No	0 (0.0%)	1 (12.5%)	15 (20.8%)	13 (15.3%)	42 (20.9%)	35 (28.7%)	<0.001
	Yes	0 (0.0%)	5 (62.5%)	41 (56.9%)	68 (80.0%)	148(73.6%)	73 (59.8%)	
	I don’t know	2 (100.0%)	2 (25.0%)	16 (22.2%)	4 (4.7%)	11 (5.5%)	14 (11.5%)	
**Section V—Dental History**	**Item 17—“Has the child ever visited a dentist?”**							
	No	1 (50.0%)	2 (25.0%)	28 (38.9%)	37 (43.5%)	52 (25.9%)	28 (23.0%)	0.007
	Yes	1 (50.0%)	6 (75.0%)	44 (61.1%)	48 (56.5%)	149(74.1%)	94 (77.0%)	
	**Item 18—“If yes, at what age was the first visit?”**							
	Mean ± SD	5	4 ± 1.41	3.24 ± 1.28	3.39 ± 1.19	2.93 ± 1.64	2.82 ± 1.85	0.002
	Median (IQR)	5	4 (2.7–5.2)	3 (2–4)	3 (3–4)	3 (1.7–4)	2 (2–3.2)	
	**Item 19—“What was the main reason for the first visit?”**							
	Check-up	0 (0.0%)	2 (33.3%)	21 (47.7%)	34 (70.8%)	102(68.5%)	69 (73.4%)	0.001
	Frontal teeth caries	0 (0.0%)	0 (0.0%)	2 (4.5%)	2 (4.2%)	18 (12.1%)	4 (4.3%)	
	Dental pain	1 (100.0%)	4 (66.7%)	12 (27.3%)	12 (25.0%)	19 (12.8%)	15 (16.0%)	
	Dental trauma	0 (0.0%)	0 (0.0%)	6 (13.6%)	0 (0.0%)	8 (5.4%)	5 (5.3%)	
	Posterior teeth caries	0 (0.0%)	0 (0.0%)	2 (4.5%)	0 (0.0%)	2 (1.3%)	0 (0.0%)	
	Extraction	0 (0.0%)	0 (0.0%)	1 (2.3%)	0 (0.0%)	0 (0.0%)	0 (0.0%)	
	Other surgical intervention	0 (0.0%)	0 (0.0%)	0 (0.0%)	0 (0.0%)	0 (0.0%)	1 (1.1%)	
	**Item 20—“Has your child had dental caries so far?”**							
	No	1 (50.0%)	1 (12.5%)	35 (48.6%)	56 (65.9%)	112(55.7%)	72 (59.0%)	0.054
	Yes	1 (50.0%)	7 (87.5%)	34 (47.2%)	29 (34.1%)	86 (42.8%)	46 (37.7%)	
	I don’t know	0 (0.0%)	0 (0.0%)	3 (4.2%)	0 (0.0%)	3 (1.5%)	4 (3.3%)	
	**Item 21—“If yes, at what age were the first caries identified?”**							
	Mean ± SD	5	3.93 ± 1.64	3.81 ± 1.22	3.72 ± 1.4	3.61 ± 1.38	3.73 ± 1.18	0.840
	Median (IQR)	5	4 (3–6)	4 (3–5)	3 (3–5)	3.5 (3–4.6)	4 (3–5)	
	**Item 22—“How many carious lesions does your child currently have?”**							
	I don’t know	1 (50.0%)	1 (12.5%)	7 (9.7%)	1 (1.2%)	7 (3.5%)	7 (5.7%)	0.002
	None	1 (50.0%)	1 (12.5%)	39 (54.2%)	67 (78.8%)	135(67.2%)	87 (71.3%)	
	1–3	0 (0.0%)	4 (50.0%)	19 (26.4%)	13 (15.3%)	47 (23.4%)	23 (18.9%)	
	4–6	0 (0.0%)	2 (25.0%)	7 (9.7%)	3 (3.5%)	10 (5.0%)	4 (3.3%)	
	More than 6	0 (0%)	0 (0%)	0 (0%)	1 (1.2%)	2 (1%)	1 (0.8%)	
**Section VI—Additional Information**	**Item 23—“Have you received information about the prevention of dental caries in children?”**							
	No	1 (50.0%)	0 (0.0%)	12 (16.7%)	3 (3.5%)	18 (9.0%)	15 (12.3%)	0.010
	Yes, from other sources	0 (0.0%)	3 (37.5%)	16 (22.2%)	17 (20.0%)	41 (20.4%)	24 (19.7%)	
	Yes, from the dentist	1 (50.0%)	5 (62.5%)	38 (52.8%)	64 (75.3%)	117(58.2%)	68 (55.7%)	
	Yes, from both	0 (0.0%)	0 (0.0%)	6 (8.3%)	1 (1.2%)	25 (12.4%)	15 (12.3%)	
	**Item 24—“Would you like to participate in a short information session on children’s oral health?”**							
	No	0 (0.0%)	3 (37.5%)	17 (23.6%)	4 (4.7%)	41 (20.4%)	37 (30.3%)	<0.001
	Yes	2 (100.0%)	5 (62.5%)	55 (76.4%)	81 (95.3%)	160(79.6%)	85 (69.7%)	

## Data Availability

The data presented in this study are available upon request from the corresponding author. The data are not publicly available due to privacy reasons.
